# Voluntary counseling and testing for HIV among high school students in the Tiko health district, Cameroon

**Published:** 2012-09-24

**Authors:** Eposi Christiana Haddison, Georges Nguefack-Tsagué, Michel Noubom, Wilfried Mbatcham, Peter Martins Ndumbe, François-Xavier Mbopi-Kéou

**Affiliations:** 1 Faculté de Médecine et des Sciences Biomédicales, Université de Yaoundé I, Yaoundé, Cameroon; 2 Laboratoire National de Santé Hygiène Mobile, Ministère de la Santé Publique, Yaoundé, Cameroon; 3 Department of Biochemistry, Laboratory of molecular Biology, University of Yaounde I, Cameroon; 4 Faculty of Health Sciences, University of Buea, Cameroon

**Keywords:** HIV, VCT, knowlegde, attitudes, adolescents, youths, students

## Abstract

The objective of this study was to evaluate the use of voluntary counseling and testing (VCT) services for HIV by high school students in the Tiko health district (THD), Cameroon. A cross sectional descriptive, analytical study was conducted using a pre-established questionnaire among high school students in the Tiko health district where a multi stage sampling method was used. A total of 474 students were included in the study. Among them, 350 (73.8%) had heard about VCT, 136 (27.8%) had undergone VCT and 329 (69.4%) were willing to undergo VCT. The use of VCT services was positively associated with age (p<0.001), sex (p<0.001), school (p<0.001), sexual activity (p=0.001), attitude (p=0.001) towards and knowledge of VCT (p<0.001). Knowledge of VCT among the students was high but the use of VCT services was low. We recommend that free screening for HIV should be offered in secondary schools of THD.

## Introduction

Voluntary counseling and testing (VCT) is a major component of HIV prevention and care. VCT is also perceived to be an effective strategy in risk reduction among sexually active young people [1]. A recent study in Cameroon reported an HIV prevalence of 3.2% [2] among youths aged 15-24 and an estimated 4.3 to 5.9 million young people in the same age group living with HIV [3]. The Cameroonian government has done a lot to reduce the incidence of HIV infection [4]. However, the Global AIDS epidemic report 2008 indicated that only 16% of the Cameroonian population aged 15-49 knew their HIV status [5]. Another study in Cameroon showed that awareness and use of centres offering VCT for HIV were very low [6]. The aim of this study was to evaluate the use of VCT services among high school students in the Tiko health distrit (THD).

## Methods

This was a descriptive cross sectional study conducted in October 2011 in order to assess knowledge, attitude and practices towards VCT among high school students in Tiko, a rural area situated in the South West Region of Cameroon. The sample size of the study determined using a single population proportion formula [7] was 232. To eliminate the design effect the sample size was doubled and 10% non-response rate was added giving a sample size of 500. The questionnaires were administered anonymously to the students in class during their break period. Data analysis was performed using Epi Info statistical software version 3.5.1. Significant level was set at p<0.05 and a 95% confidence interval was also set.

## Results

This study included 474 students among which 235 (49.5%) were boys and 239 (50.5%) girls. Their ages ranged from 14-25 years with a mean age of 18.3 ± 1.8 years (Table 1). Two hundred and thirty three (49%) students were in Lower sixth and 241 (51%), in Upper sixth. All the respondents had heard about HIV/AIDS with their main sources of information being school (61.5%) and the media (50.5%). The three main modes of transmission identified were; unprotected sexual contact (82.5%), contaminated blood transfusions (69.8%) and contaminated sharp objects (60.1%). According to respondents, the means of prevention of HIV/AIDS were abstinence (mentioned by 95.4, use of condoms (mentioned by 48.3%) and faithfulness to one partner (mentioned by 46.4%). Most of the respondents (73.8%) had heard about VCT with their main sources of information being television (40.3%) and school (34%). The majority (82.8%) of those who had heard of VCT identified health institutions as VCT providers, while 48.8% identified counseling and 66.2% testing, as activities carried out at VCT services. Four hundred and twelve (86.9%) respondents reported that VCT was necessary with as main reason, the fact that it made them know their HIV status (80%). The majority (83.1%) of the students also reported that everybody should go for VCT and 78.9% reported they could recommend VCT to a family member. Two hundred and twenty four (47.3%) respondents were sexually active. The mean age of sexual debut was 16.9 ± 1.7 years. More than 60% of them (64.7%) had had sex before they were 18 years with 22.3% having their sexual debut at 15 years or less. Also, 42.8% of the sexually active population had had two or more sexual partners in the last year while 59% had had unprotected sex. However, only 18.8% of the students felt they were at risk of getting HIV.

**Table 1:** Socio-demographic characteristics of the study population

Characteristics	Total	
	Number (N=474)	Percentage ( %)
**Sex**		
Male	235	49.5
Female	239	50.5
Government	213	45
Lay Private	151	32
Mission	110	23
**Class**		
Lower sixth	233	49
Upper sixth	241	51
**Religion**		
Christianity	467	98.5
Islam	5	1
Others	2	0.5
**Age**		
14-16	63	13.3
17-19	304	64.1
20-22	96	20.3
23-25	11	2.3

Only 136 (27.8%) students had used VCT and this was significantly lower than the 80% objective set by the Cameroon government (p<0.001). An association was found between VCT attendance and knowledge of VCT (p=0.001). Compared to younger students (14-16 years), students aged 23-25 years were 7 times (OR=7.2 CI 1.81, 28.6) more likely to have attended a VCT service. Females were 3 times (OR= 2.97, CI 1.99, 4.66) more likely to have gone for VCT than males (Table 2). Compared to students from government schools, students from lay private schools were 2.3 times (OR=2.34, CI 1.49, 3.66) more likely to have attended a VCT service. Students who said VCT was necessary were 14 times (OR=14.4, CI 3.4, 86.8) more likely to have gone for VCT than those who thought it was not necessary. Students who said everybody should attend VCT services were 3 times (OR=2.91, CI 1.46, 6.31) more likely to have gone for VCT than those who did not. Finally, compared to students who had never had sex before, students who were sexually active were 2 times (OR= 1.92, CI 1.26, 2.94) more likely to have gone for VCT.

**Table 2:** Relationship between attented a VCT service and selected characteristics

Characteristics	Attended a VCT service			UnadjustedOR (95% CI)	Adjusted OR (95% CI)	P value
	Yes (%)	No (%)	TotalN=474			
**Age**						
14-16*	9 (14.3)	54 (85.7)	63	1	1	
17-19	79 (26)	225 (74)	304	2.10 (0.99,4.46)	1.63(0.72,3.67)	0.05
20-22	2 (43.8)	54 (56.3)	96	4.66 (2.04,10.5)	2.49(0.94,6.56)	<0.001
23-25	6 (54.5)	5 (45.5)	11	7.20 (1.81,28.6)	4.32(0.92,20,3)	<0.001
**Sex**					P value for trend	<0.001
Male*	42 (17.9)	193 (82.1)	235	1	1	
Female	94 (39.3)	145 (60.7)	239	2.97(1.99, 4.66)	3.33 (1.92,5.0)	<0.001
**School**						
Government*	52 (24.4)	161 (75.6)	213	1	1	
Lay private	65 (43)	86 (57)	151	2.34(1.49, 3.66)	1.52(0.97,2.26)	<0.001
Mission	19 (17.3)	91 (82.7)	110	0.64(0.36, 1.16)	0.67(0.35,1.28)	0.14
**Class**					P value for chi-square	<0.001
Lower sixth*	60 (25.9)	172 (74.1)	233	1	1	
Upper sixth	76 (31.4)	166 (68.6)	241	1.31 (0.86, 2.0)	1.16(0.72,1.88)	0.18
**Is VCT necessary**						
No*	2 (3.2)	60 (96.8)	62	1	1	
Yes	134 32.5)	278 (67.5)	412	14.4 (3.4,86.8)	12.4(2.89,53.3)	<0.001
**Should everybody attend VCT**						
No*	11 13.8)	69 (86.3)	80	1	1	
Yes	125 31.7)	269 (68.3)	394	2.91 (1.46,6.31)	2.07(0.99,4.34)	0.001
**Sexually active**						
No*	56 (22.4)	194 (77.6)	224	1	1	
Yes	80 (35.7)	144 (64.3)	250	1.92 (1.26,2.94)	1.83(1.12,2.97)	0.001
**At risk of getting HIV**						
No*	107 27.8)	278 (72.2)	89	1	1	
Yes	29 (32.6)	60 (67.4)	385	1.25 (0.73,2.11)	1.21(0.68,2.14)	0.44

* Reference level for computing Odd ratios (ORS)

## Discussion

All respondents had heard about HIV/AIDS with their main sources of information being school (61.5%) and the media (50.5%). The percentage of students who had heard about HIV/AIDS is similar to those obtained by Tarkang in Kumba, Cameroon (95.8%) [8]. This shows that awareness of HIV/AIDS amongst youths in Cameroon is high. In this study, 73.8% of the respondents had heard about VCT. These figures are lower than those obtained by Abiy in Ethiopia and Muganda in Kenya whereby 96.7% and 92% respectively of their study populations had heard about VCT [9, 10]. Most of the respondents (86.9%) reported that VCT was necessary. These results are similar to those obtained by Omary in Tanzania (95.8%) [11]. A majority of the respondents (83.1%) said everybody should attend VCT services and 78.9% said they could recommend VCT to a family member.

Two hundred and twenty four (47.3%) students were sexually active. This result is similar to that obtained in Kumba, Cameroon (58%) [8], but greater than those in other studies reported elsewhere in Africa [9, 10]. This high rate of in-school sexual activity in Cameroon as compared to other African countries may be due to the fact that there are no implementation of rules prohibiting youths from doing as they like such as, buying and consuming cigarettes, alcohol or going to night clubs before their majority and these activities are associated with sexual activity. About 42.8% of the sexually active population had had two or more sexual partners in the last year while 59% had had unprotected sex. Only 18.8% of the respondents felt they were at risk of getting HIV/AIDS. This result is similar to that obtained by Mitike in Ethiopia (19%) [11]. This low perceived susceptibility may be due to the fact that youths feel invincible to HIV/AIDS and may explain why they still engage in risky sexual behaviours.

Despite the high awareness of VCT services (73.8%) among the students, less than a third (28.7%) of them had gone for VCT with more females than males. This result is similar to those obtained in other African countries [11, 13]. The main reason given for using VCT services by those who had done so was to know their HIV status (94.8%). A similar result was obtained in Tanzania [11]. Those who had never had VCT gave as main reason the fact that they did not know where to go (50.5%). Another reason given for not using VCT services was that they did not think it was necessary for them (34%). This result is similar to that obtained in Tanzania where 34% of the respondents who had never had VCT felt it was not meant for students [11]. Concerning the future use of VCT services, more than half (69.4%) of the respondents said they would like to be tested for HIV. This finding is encouraging when compared to the 28.7% of respondents who had had VCT. However, this finding is lower than that found in other African countries (Nigeria 84.6%, Ethiopia 82.5%) [9, 14]. Concerning factors that influence VCT uptake, we see that with age, the students were more likely to have gone for VCT. This may be due to the fact that as age increases, the students become more mature and are able to better assimilate HIV information. Students from lay private schools were more likely to have used VCT services than their peers from other schools. In mission schools talking about sex is taboo since premarital sex is forbidden by Christianity, so we would expect a very low rate of VCT utilisation among the students because anyone going for VCT is considered sexually active. The findings of this study must be interpreted in the context of the limitations encountered. Amongst these limitations was the fact that in this study, we mostly relied on information provided by the respondents of our questionnaire. In addition, our study was a single district survey and the results may not be generalised to the majority of high school students in Cameroon.

## Conclusion

Knowledge of HIV and VCT among the students was high and they had a positive attitude towards VCT, however, the use of VCT services was low. VCT attendance was influenced by age, sex, school, sexual activity, attitude towards and knowledge of VCT. We therefore recommend that sensitisation campaigns on HIV should continue with emphasis being laid on VCT and free HIV screening in secondary schools in the Tiko Health District of Cameroon.
